# A Comparison of Target Gene Silencing using Synthetically Modified siRNA and shRNA That Express Recombinant Lentiviral Vectors

**Published:** 2009-07

**Authors:** P.V. Spirin, D. Baskaran, P.M. Rubtsov, M.A. Zenkova, V.V. Vlassov, E.L. Chernolovskaya, V.S. Prassolov

**Affiliations:** 1 Engelghardt Institute of Molecular Biology, Russian Academy of Sciences;; 2 Institute of Chemical Biology and Fundamental Medicine, Siberian Branch, Russian Academy of Sciences

## Abstract

RNA-interference is an effective natural mechanism of post-transcriptional modulation of gene expression. RNA-interference mechanism exist as in high eukaryotes both animals and plants as well in lower eukaryotes and viruses. RNA-interference is now used as a powerful tool in study of functional gene activity and many essential for fundamental biology results was obtained with this approach. Also it's widely believed that RNA-interference could be used in working out of new therapeutic medicine against malignant, infectious and hereditary diseases. One of the main problems of these developments is search of effective methods of siRNA transfer into the target cells. At present time for these purpose different sorts of transfect ions or viral transduction are used. At present article the results of comparison of inhibition of expression of oncogene AML-ET O by synthetic siRNA and by recombinant lentiviruses coding for corresponding shRNA are presented.

## INTRODUCTION

The controlled silencing of target genes is important both for molecular biological studies and for related applied sciences: in particular, modern biomedicine.

Among the many gene silencing approaches (which include the use of anti-sense RNA, ribozymes, chemical blockers, and targeted mutagenesis), the most efficient approach is based on RNA-interference.

RNA interference is a highly specific mechanism for the posttranscriptional silencing of target genes. It involves the degradation of the target gene mRNA. The degradation of mRNA occurs in a complex formed by short-interfering RNA oligonucleotides (siRNA) and cellular proteins such as endonucleases. The nucleotide sequence of siRNA is complementary to that of target gene mRNA.

In the past couple of years, the use of siRNA has become widespread in studies of gene functioning and gene interaction. The use of siRNA as next generation therapeutic agents in biomedicine is also being explored. It is possible that, in the near future, siRNA will be used for treating viral and oncological diseases.

Currently, short synthetic 21-22-bp double-stranded siRNA molecules are widely used to silence mammalian genes. A number of commercial firms synthesize siRNA oligonucleotides. These commercial firms have siRNA design tools available on their websites (e.g., www.qiagen.com). Synthetic siRNA oligonucleotides are transferred into cells in vitro by lipofection. Since siRNA induces the degradation of mRNA (and not the protein directly), the silencing effect does not occur immediately after cell transfection. The silencing effect is generally noticeable within 18 hours of transfection: however, in the case of stable proteins, the silencing effect may be noticeable only 24-48 hours after transfection. The longevity of siRNA silencing is comparatively short, and different sources claim that the silencing effect lasts for 3-5 cell divisions. It should be noted that the longevity of siRNA silencing may depend on many factors, in particular the nature of the cells being transfected. Approaches have been developed to synthetically modify siRNA oligonucleotides, which enhance the longevity of siRNA silencing in cells. Such synthetically modified siRNA oligonucleotides are useful for the post-transcriptional silencing of genes that encode proteins with a long half-life.

For long-lasting gene silencing, shRNA expressing lentiand retroviral vectors can be used. Nucleotide sequences encoding the sense and antisense strands of siRNA separated by a spacer sequence can be cloned into lenti- or retroviral vector constructs using standard molecular biological cloning techniques. The transcription of such nucleotide sequences leads to the formation of shRNA molecules. shRNA molecules form a hairpin structure consisting of two complimentary strands separated by a loop (spacer). The cellular endonuclease dicer is responsible for the cleavage of shRNA molecules. As a result, the loop (spacer) gets removed from shRNA molecules and double-stranded siRNA molecules are formed. These siRNA molecules are capable of initiating the degradation of target gene mRNA.

Within the framework of our project, we were able to silence the expression of activated oncogenes AML1-ETO (t8;21) and RUNX1(K83N) with the help of RNA interference. These activated oncogenes are frequently found in acute myeloid leukemia patients. We were able to compare the efficiency of gene silencing (a) after the lipofection of oncogene-expressing model cell lines with synthetically modified double-stranded siRNA oligonucleotides and (b) after the transduction of oncogene-expressing model cell lines with shRNA-expressing recombinant lentiviral vector particles.

Oncogene-expressing model cell lines were obtained from murine SC1 embryonic fibroblast cell lines after their transduction with bicistronic retroviral vector particles. These retroviral vectors contained a bicistronic expression cassette comprised of the gene of interest and an eGFP marker gene separated by an IRE S sequence and driven by a common promoter. The following genes were selected as genes of interest:

(1) The AML1-ETO fusion gene, which is formed as a result of the t(8:21) chromosomal translocation.

(2) The activated RUNX1-K83N oncogene, which is formed as a result of a point mutation in the RUNX1 gene and leads to the substitution of lysine to asparagine in the 83 position of the RUNX1 protein. Since both the gene of interest and eGFP marker gene are driven by a common promoter, the expression levels of the gene of interest in cells can be evaluated based on the intracellular expression of the eGFP marker gene.

**Fig. 1.**
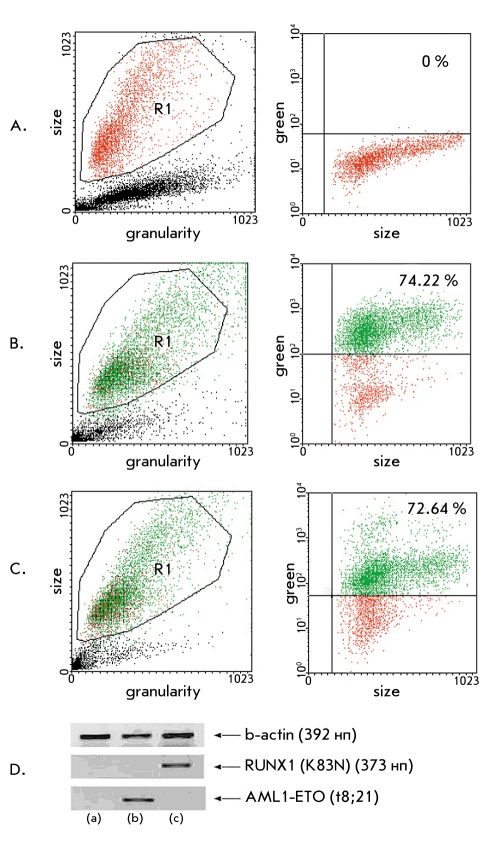
Efficiency of transgene expression in cell lines transduced with AML1-ETO- and RUNX1-K83N-expressing recombinant retroviral vector particles; (A) Histogram of nontransduced SC-1 murine fibroblast cell line, (B) Histogram of SC-1 murine fibroblast cell line transduced with RUNX1-K83N expressing retroviral vector particles (74,22 % of the population shows increased fluorescence), (C) Histogram of SC-1 murine fibroblast cell line transduced with AML1-ETO expressing retroviral vector particles (72,64 % of the population shows increased fluorescence). The absolute values of the size of each event analyzed (cell) is shown along the X axis, while the absolute values of the intensity of fluorescence of each event (cell) is shown along the Y axis. Every third histogram in each row is divided into 4 quadrants; the upper right quadrant contains cells expressing eGFP and AML1-ETO, while the lower right quadrant contains cells that do not express eGFP and AML1-ETO. (D) RT-PCR (from left to right; (a) non transduced SC-1 cell line, (b) SC-1 cell line transduced with RUNX1-K83N expressing retroviral vector particles, (c) SC-1 cell line transduced with AML1-ETO expressing retroviral vector particles)

The typical results of transduction efficiency and transgene expression are shown in Fig. 1.

Throughout the course of our work, we used the murine SC1 embryonic fibroblast cell line, the HEK 293 cell line, and two transgenic SC1 cell lines expressing either activated oncogene AML1-ETO or RUNX1(K83N). All four cell lines were cultured in standard DMEM medium containing 10% FBS, 4mM L-glutamine, 1mM sodium pyruvate, 100 mkg/ ml streptomycin, and 100 units/ml penicillin at 37 ≤C in a 5% CO2 atmosphere.

The design and synthesis of double-stranded siRNA molecules was carried out in collaboration with the Institute of Chemical Biology and Fundamental Medicine, Siberian Branch of the Russian Academy of Sciences (ICBFM SB RAS). The oligonucleotides were synthesized at ICBFM SB RAS using the phosphamide method. During oligonucleotide synthesis, a unique method was used to methylate the oligonucleotides at certain positions. These modifications significantly increase the lifespan of synthetic siRNA in cells. The nucleotide sequences of siRNA oligonucleotides and the oncogene mRNA zones being targeted by them are shown in Table. 1.

**Table 1:** Table 1

target	chain	sequence
AML-ETO	sense	5'-CCUCGAAAUCGUmACUmGAG**U**AG-3'
	antisense	5'-UCUCmAGUmACGAUUUCGAGGUU-3'
ETO	sense	5'-GGCCmAGCGGUmACmAGUCCmA**G**AU-3'
	antisense	5'-UUmGGACUmGUmACCGCUmGGCCUG-3'
AML(5')	sense	5'-GAACCmAGGUUmGCmAAGAUU**G**AA-3'
	antisense	5'-AAAUCUUmGCmAACCUmGGUUCUU-3'
AML(3')	sense	5'-AGCCCGGGAGCUUmGUCCU**C**UU-3'
	antisense	5'-AAGGACmAAGCUCCCGGGCUUmG-3'

Anti-AML1-ET O and anti-RUNX1 siRNA duplexes were transferred into transgenic SC1 cell lines by lipofection using Lipofectamin2000 (Invitrogen) as per the manufacturers' protocol. The final concentration of siRNA duplexes in the transfection culture medium was 200nM. Four hours after transfection, the culture medium was changed to a fresh medium. The transfected cell lines were cultured at 37 ≤C in a 5% CO2 atmosphere for 72 hours.

The level of oncogene expression in the cell lines was evaluated by reverse transcription PCR (RT -PCR ) and flow cytometry. The total mRNA from the cell lines was extracted using Trizol (Invitrogen) as per the manufacturers' protocol. The extracted mRNA was used to synthesize the first strands of cDNA using the ImProm-II™ Reverse Transcriptase (Promega) kit. 

PCR with sequence-specific primers was carried out to identify the nucleotide sequences of the genes of interest and eGFP marker gene in the total cDNA. The following primers were used for RUNX1(K83N): sense-AGTCTACCAATACCTGGGA; antisense-TCTCAGCTGTGGTGGTGAAG, for AML1/ET O: sense-CATTCACCGAGATAGGAG; antisense-AAGTCTCGGCGTCACTGAT, for eGFP : sense-ACCTACGGCCTGCAGTGCT; antisense-TGCCGTTCTCTGCTGTCG. PCR products were then visualized after 1.5% agarose gel electrophoresis. The results were processed using the Gel-Pro Analyzer 4.0 software, which gives a maximum optical density (maxOD) reading. The results were normalized against ≤-actin.

**Fig. 2.**
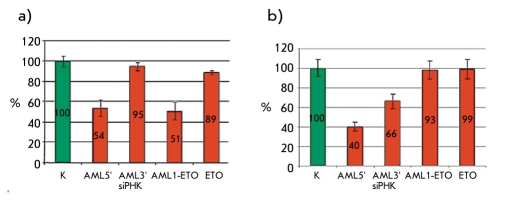
Results obtained after flow cytometric analysis of cell suspensions. The percentage of fluorescent cells in the population of different siRNA transfected cell lines is shown. A) The percentage of fluorescent cells in the AML1-ETO expressing murine fibroblast cell line 48 hours after transfection with various siRNA duplexes. B) The percentage of fluorescent cells in the RUNX1(K83N) expressing murine fibroblast cell line 48 hours after transfection with various siRNA duplexes

Cell fluorescence was measured using an Epics 4XL Beckman Coulter flow cytometer (United States). The WinMDI2.8 software program was used for data collection and data analysis. The diagrams of the percentage of fluorescent cells in the cell populations are shown in Fig. 2. Cell populations transfected with respective siRNA duplexes are shown along the X axis, while the percentage of fluorescent cells in the population is shown on the Y axis.

Results obtained by flow cytometry correlate well with the results obtained by RT-PCR . The electrophoregram of RT-PCR products is shown in Fig. 4. The reduction of oncogene mRNA is clearly visible in the SC1-AML1/ET O(t8;21) transgenic cell lines transfected with siRNA oligonucleotides targeting the 5'-end of RUNX1 mRNA, ETO mRNA and the site of junction of AML1-ETO mRNA. Oncogene mRNA reduction is also seen in the SC1-RUNX1(K83N) transgenic cell lines transfected with siRNA oligonucleotides targeting the 5'- and 3'-ends of RUNX1 mRNA.

**Fig. 3.**
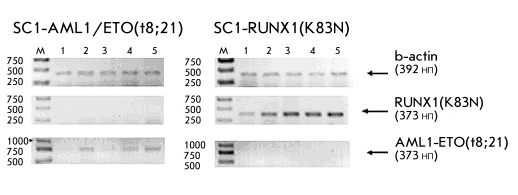
The electrophoregram of RT-PCR products of total cDNA obtained from oncogene expressing model cell lines transfected with siRNA. 1) siRNA(AML1-5`), 2. siRNA(AML1-3`), 3. siRNA(AML1-ETO), 4. siRNA(ETO), 5. Control (without siRNA)

**Fig. 4.**
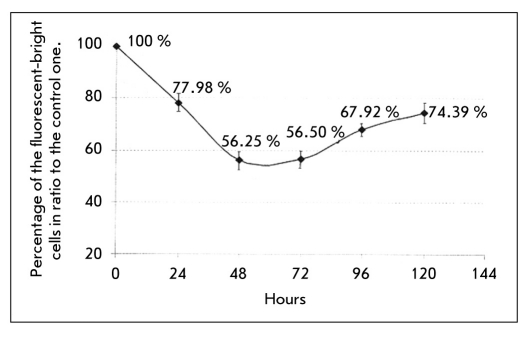
The gene silencing kinetics of synthetic siRNA oligonucleotides targeting the junction point of AML1-ETO mRNA

While there is a significant reduction of fluorescence in a portion of the cell population (two times or more) after the addition of synthetic siRNA, there is no such reduction in fluorescence in the rest of the cell population (Fig. 3). This coincides with the results obtained by RT-PCR (Fig. 3). The most likely reason for this is the low efficiency of siRNA delivery into cells. According to the manufacturers of Lipofectamin2000 (Invitrogen), the lipofection efficiency with siRNA oligonucleotides is 50-60% and depends on the nature of the cells being transfected. Apart from this, there is the possibility of a reduction of synthetic siRNA activity due to their enzymatic degradation, which is catalyzed by cellular endonucleases, despite the stabilizing modifications introduced to the siRNA oligonucleotides. According to our data, the maximum interfering activity of synthetically modified siRNA is observed 48-72 hours post transfection. The gene silencing kinetics of synthetic siRNA oligonucleotides targeting the junction point of AML1-ETO mRNA is illustrated in Fig. 4. The moment of siRNA transfection has been taken as zero-time reference.

It has been shown that the maximum silencing activity of synthetic siRNA is 48-72 hours post transfection, when the percentage of fluorescent cells in the population is around 56%. The percentage of fluorescent cells in the population increases to 67.92 and 74.39% 96 and 120 hours post transfection, respectively. The results shown are averaged from three parallel measurements and are in reference to a control. The efficiency of RNA-interference can be increased by increasing the efficiency of siRNA delivery into the model cell lines. For this purpose, taking into account the efficiency of the synthetic siRNA duplexes, two shRNA-expressing lentiviral vectors were constructed targeting the junction point of AML1-ETO mRNA and the 5`-end of RUNX1 mRNA, respectively. The lentivectors were constructed by cloning the shRNA-expressing DNA sequence into the pLSLP vector.

DNA sequence which encodes anti AML1-ETO shRNA

AML1-ET O-sense

5'-p-gatccgCCTCGAAATCGTACTGAGGcttcctgtcaTCTCAGTACGATTCGAGGtttttg-3'

AML1-ET O antisense

5'-p-aattcaaaaaCCTCGAAATCGTACTGAGAtgacaggaagCCTCAGTACGATTCGAGGcg-3'

DNA sequence which encodes anti-RUNX1 shRNA

AML-5-sense

5'-p-gatccgGAACCAGGTTGCAAGATTCcttcctgtcaAAATCTGCAACCTGGTTCtttttg-3'

AML-5-antisense

5'-p-aattcaaaaaGAACCAGGTTGCAAGATTtgacaggaagGAATCTGCAACCTGGTTCcg-3'

**Fig. 5.**
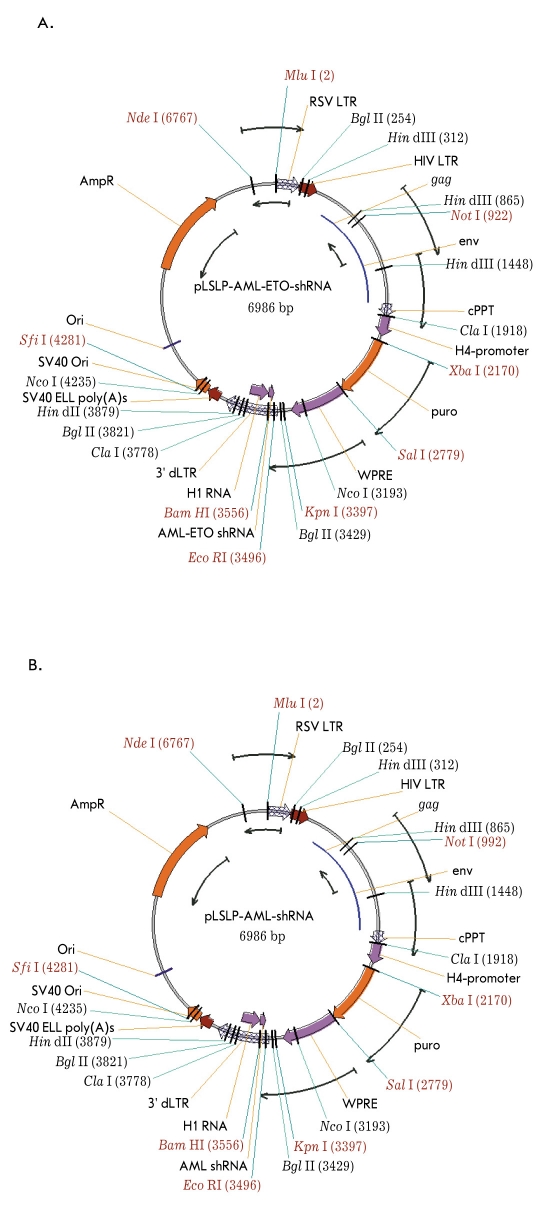
shRNA expressing lentiviral vectors; (A) pLSLP-AML-ETO-shRNA: lentiviral vector expressing shRNA targeting the junction point of AML1-ETO mRNA, (B) pLSLP-AML-shRNA: lentiviral vector expressing shRNA targeting the 5`-end of RUNX1-K83N mRNA

The design of the shRNA-coding DNA sequences was done through an internet resource (http://gesteland.genetics.utah.edu/siRNA_scales/index.html). Standard genetic engineering techniques were used for all cloning procedures. The vector maps of the constructed shRNA-expressing lentiviral vectors are shown in Fig. 5.

The transient cotransfection of HEK293 T cells with shRNA-expressing lentiviral vectors and packaging plasmids was done for the production of shRNA-expressing lentiviral vector particles. The transfection was carried out using Lipofectamin2000 (Invitrogen) as per the manufacturers' protocol. Viral stocks were harvested for three days and used for the infection/transduction of the transgenic model cell lines expressing the activated oncogenes AML1-ETO and RUNX1(K83N). Forty-eight hours after transduction/infection, the model cell lines were placed in culture media containing puromycin (10mkg/ml) for five days. After selection for puromycin resistance, the shRNAexpressing transduced model cell lines were analyzed by flow cytometry.

**Fig. 6.**
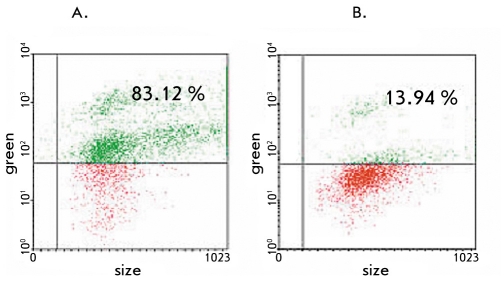
Histogram illustrating the reduction in fluorescence in the AML1-ETO expressing SC-1 murine fibroblast cell line: (A) before transduction, (B) after transduction with shRNA-expressing lentiviral vector particles targeting the junction point of the AML1-ETO mRNA

**Fig. 7.**
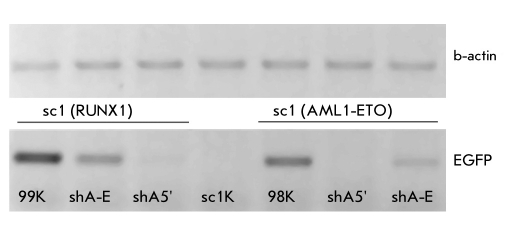
The electrophoregram of RT-PCR products of total cDNA obtained from RUNX1(K83N) and AML1-ETO oncogene expressing SC1 model cell lines transduced with shRNA expressing lentiviral vector particles: 99K - control (non-transduced RUNX1(K83N) expressing SC1 cell line); shA-E - RUNX1(K83N) expressing SC1 cell line transduced with shRNA expressing lentiviral vector particles targeting the junction point of AML1-ETO mRNA; shA5` - RUNX1(K83N) expressing SC1 cell line transduced with shRNA expressing lentiviral vector particles targeting the 5`-end of RUNX1 mRNA . sc1K - control (non transduced SC1 cell line); last three samples - Analysis of AML1-ETO(t8;21) expressing SC1 cell lines (98k - non transduced control, shA-E, sh5` - transduced with corresponding lentiviral vector particles)

The percentage of fluorescent cells in the SC1-AML1/ETO(t8;21) transgenic cell line decreased 6 times after their transduction with shRNA-expressing lentiviral vector particles targeting the junction point of AML1-ETO mRNA (Fig. 6). Similar results were seen when the SC1-AML1/ETO(t8;21) and SC1-RUNX1(K83N) cell lines were transduced with shRNA-expressing lentiviral vector particles targeting the 5`-end of RUNX1 mRNA. The percentage of fluorescent cells in the cell lines decreased 8 and 8.5 times, respectively. It should be noted that the SC1-RUNX1(K83N) cell line showed no reduction in fluorescence when transduced with shRNA-expressing lentiviral vector particles targeting the junction point of AML1-ETO mRNA. The results obtained by flow cytometry correlate well with the results obtained by RT-PCR (the electrophoregram is shown in Fig. 7).

## CONCLUSIONS

Our results illustrate the high efficiency of stable synthetically modified siRNA duplexes for silencing the activated oncogenes frequently found in acute myeloid leukemia patients. Validated siRNA sequences were used to design and synthesize shRNA-coding DNA sequences. The synthesized DNA sequences were cloned into a recombinant lentiviral vector. The shRNA-expressing lentiviral vector particles targeting the junction point of AML1-ETO mRNA and 5`-end of RUNX1 mRNA were used to transduce the oncogene-expressing model cell lines. The transduced model cell lines were analyzed by RT-PCR and flow cytometry. The analysis revealed a significant reduction in the expression of activated oncogenes in the transduced cell lines. This is indicative of the high efficiency of the constructed lentiviral vector constructs, which can be used for silencing target genes by RNA interference.

## Acknowledgements

We would like to thank Prof. P.M. Chumakov for providing us with the pLSLP lentiviral vector. The experimental work was carried out within the framework of the "Molecular and Cell Biology" project and project No. 27 "Fundamental Research Basics in Nanotechnology and Nanomaterials" of the RAS presidium fundamental research program. 
